# Happy for Us not Them: Differences in neural activation in a vicarious reward task between family and strangers during adolescent development

**DOI:** 10.1016/j.dcn.2021.100985

**Published:** 2021-06-30

**Authors:** Philip Brandner, Berna Güroğlu, Suzanne van de Groep, Jochem P. Spaans, Eveline A. Crone

**Affiliations:** a Erasmus School of Social and Behavioural Sciences, Erasmus University Rotterdam, the Netherlands; b Leiden Institute for Brain and Cognition, Leiden University, the Netherlands

**Keywords:** Adolescence, Vicarious reward, Nucleus accumbens, Family relationships, Cooperation

## Abstract

During adolescence social-interactions with other people become more relevant. One key aspect of these interactions is cooperative behavior. Cooperation relies on a set of cognitive and affective mechanisms. In this study, we focused on the mental ability to feel happy for another person’s positive experience, called vicarious joy. We investigated the neural mechanisms of this ability using a false-choice vicarious reward fMRI task. Participants played a game where they could win monetary rewards for themselves, their mother, their father, and a stranger. A region-of-interest (ROI) analysis of the Nucleus Accumbens revealed robust activation in this region for personal reward as well as vicarious rewards for both parents. Vicarious reward for a stranger was not associated with activation within the Nucleus Accumbens. ROI activation was associated with self-reported vicarious joy for mother and father. A Prisoner’s Dilemma game outside the scanner showed an increase in cooperative behavior until age 14 for parents and strangers, followed by a decline for the stranger but not for the parents. Together, these findings demonstrate that adolescence is an important time for developing ingroup-outgroup relations.

## 1 Introduction

Adolescence is an important transition period for social, cognitive, and affective development (Crone and Dahl, 2012; van den Bos et al., 2011). During this time, children mature from having ego-centric motivations to incorporating other people’s perspectives and goals into their decision-making (Nelson and Guyer, 2011). Prior studies demonstrated that cooperation, an important behavior for building social relations, becomes more focused on in-group versus out-group targets during adolescence (Güroğlu et al., 2014). Given the stronger in-group focus in adolescence and with most studies looking at peer-relationships, an important question that remains to be investigated is whether cooperation also changes in parent-child relationships during adolescence (Mayseless et al., 1988; Noack and Puschner, 1999; Steinberg, 2001). In this study, we examined developmental differences in cooperation with parents, and we tested whether neural responses to vicarious joy were predictive of cooperative behavior towards parents.

### 1.1 Vicarious joy

Being able to appreciate the emotional experience of other people is a foundational building block for cooperation and for building long-lasting relationships (Zaki and Ochsner, 2012). The ability to feel happy for other people’s positive experiences is called vicarious joy (Batson et al., 1991; Royzman and Rozin, 2006). Prior studies using neuroimaging measures demonstrated that reward processing for self and others (i.e., vicarious reward processing) has partly overlapping neural correlates but also dissociable patterns. Reward processing for self is associated with increased activity in a network of brain regions, including the ventral striatum, a region in the basal ganglia (Apicella et al., 1991, 1992; Kawagoe et al., 1998). Similar to what was previously observed in animal studies (Montague and Berns, 2002; Schultz, 2016), human studies have repeatedly found the Nucleus Accumbens (NAcc), a subregion of the ventral striatum, to be involved in reward processing (Berridge and Kringelbach, 2015; Delgado, 2007; Liu et al., 2011). The NAcc responds to a wide range of rewards, including food, social interactions, and monetary rewards (Delgado, 2007; Knutson et al., 2001; Sescousse et al., 2013) and peaks in activity in mid-adolescence (Braams et al., 2015; Schreuders et al., 2018). While the neural mechanisms underlying personal reward processing have been well studied, the neural processes for vicarious reward are less clear.

A recent meta-analysis found the ventral striatum to be predominantly activated when winning personal rewards, whereas vicarious reward activity was dependent on the beneficiary (Morelli et al., 2015). In studies where targets were close friends (Schreuders et al., 2021) or family members increased ventral striatum activity was found when receiving vicarious rewards (Braams and Crone, 2017; Fareri et al., 2012; Telzer et al., 2013; Varnum et al., 2013). These findings fit with prior studies showing that when individuals feel emotionally close to another person they are more likely to share in their positive emotional state (Mobbs et al., 2009). Furthermore, a prior study found similar activation patterns for a close friend receiving a reward compared with a personal reward but no vicarious reward response was observed when a stranger received a reward (Braams et al., 2014). In a recent study in adults, we have also shown that in the NAcc vicarious reward activation for parents is more similar to personal reward responses compared to vicarious reward activation for strangers (Brandner et al., 2020).

One additional region is consistently implicated in reward processing, the ventro-medial prefrontal cortex (vmPFC). As with the ventral striatum, the vmPFC shows a robust pattern associated with positive reward outcomes. In contrast with regions showing activation for negative as well as positive reward outcomes (dmPFC, dorsal striatum, anterior insular) the vmPFC and NAcc seem to be tuned specifically to positive effects. They are presumed to constitute a currency-independent (i.e. general) valuation system allowing for normalized subjective reward valuations across domains (Bartra et al., 2013; Clithero and Rangel, 2013). This general-purpose subjective valuation system also seems to extent to decisions regarding selfish versus prosocial choices, which mostly involves the vmPFC (Zaki et al., 2014).

### 1.2 Cooperation with related others

Cooperation among genetically related individuals (kin-selection) has been observed for a long time (Rand and Nowak, 2013). An often used paradigm to study cooperation is the Prisoner’s Dilemma Game (PDG) (Trivers, 1971). In this two-player paradigm, players are presented with two choices: they can choose to ‘cooperate’, which results in equally distributed resources among the two parties (e.g., 3 coins for both), or they can choose to ‘defect’, which gives the player the opportunity to gain a higher reward at the expense of the other player (e.g., 5 coins for self and 1 coin for other). Both players independently make a decision and the final outcome is dependent on the choices of both players. In case both players cooperate, the outcome is equal (i.e., 3 coins each). If one player cooperates and the other defects, the defecting player receives a higher reward (i.e., 5 coins) than the cooperating player (i.e., 1 coin). However, if both players defect, then they both receive nothing. Prior studies have shown that certain PDG variations make cooperation more likely to occur. First of all, cooperation depends on whether the PDG is a one-shot game or allows for repeated interactions. In one-shot games (games without repeated interactions) cooperation with unrelated strangers is relatively rare (Stevens and Hauser, 2004). On the other hand, studies based on repeated trials have shown that one strategy called tit-for-tat (TFT), seems to be quite stable across multiple runs (i.e., achieves an equilibrium within a population; Axelrod and Hamilton, 1981). These studies show that the possibility of future interactions with the same individual can promote repeated cooperative behavior. Another factor that influences PDG cooperation pertains to target (i.e., player 2) characteristics. Prior studies have demonstrated that individuals cooperate more with in-group members such as close friends or family members (Bigelow et al., 1992; Jordan et al., 2014; Padilla-Walker and Christensen, 2011). Developmental studies using prisoner’s dilemma designs showed age-related increases in cooperative behavior in adolescence (Blake et al., 2015; Gutiérrez-Roig et al., 2014). However, it is currently not yet known whether adolescents differentiate in cooperative behavior for mothers, fathers, and strangers (Güroğlu et al., 2014). Perhaps most intriguing is the question of whether vicarious reward activity may predict cooperative behavior in a prisoner’s dilemma task (Aknin et al., 2018; Kawamichi et al., 2013).

### 1.3 The current study

In the current study, we investigated the neural reward processes for personal and vicarious rewards by using a false-choice gambling task while participants underwent fMRI scanning. In this vicarious reward task participants were asked to choose between two unknown distributions of resources upon which they were presented with the outcomes of rewards for themselves and for another target, who could be their mother, father, or a stranger. In addition, we employed a behavioral prisoner’s dilemma game (outside the scanner) to allow for explicit cooperative prosocial behavior (Gutiérrez-Roig et al., 2014). This task was presented in two conditions, the classic prisoner’s dilemma condition and a second condition in which cooperative behavior was beneficial for both parties (social dilemma or snowdrift game; Doebeli and Hauert, 2005; Kummerli et al., 2007). As such, the current study allows for a novel approach investigating whether the neural correlates of vicarious reward processing are related to actual cooperative behavior towards family members and strangers. Here, we examined these processes in adolescents between ages 8–19 years to test whether vicarious rewards for parents and strangers changed across adolescence (Silverman et al., 2015). In this critical phase of social reorientation and the drive for autonomy and independence concepts of in-versus-out groups are solidified. We have therefore chosen the two target groups carefully to help improve our understanding of how adolescents perceive members of these groups at a fundamental affective level, namely vicarious reward processing. Considering the high significance of parents across adolescence, we chose to include both parents as in-group members. Strangers were chosen as an out-group member as they are most likely to be perceived in similar ways across different individuals.

This study including our methods and hypotheses was pre-registered on the open-science-framework (https://osf.io/e6fk7). Our confirmatory survey hypothesis predicted that self-reported pleasure of winning monetary rewards would be higher for family members than for strangers (*hypothesis #13*). We expected to see a higher activation in the NAcc (ROI-analysis) for rewards for the self with a quadratic peak in mid-adolescence (Braams et al., 2015); (hypothesis #15). In addition, we expected to see a higher NAcc activation for rewards for both mother and father compared to rewards for the stranger, again with a peak in mid-adolescence (Braams et al., 2014) (hypothesis #16). Furthermore, we anticipated the NAcc peak activation for each target to be positively associated with cooperative behavior towards the respective target in the PDG (hypothesis #17). We explored whether similar effects were observed for the SDG task. Although not pre-registered, based on our prior study in adults (Brandner et al., 2020), we expected that vicarious rewards in the NAcc would be larger for mothers than fathers. Additionally, we included one exploratory vmPFC ROI analysis that was not pre-registered but added valuable context of vicarious reward processing above and beyond the NAcc.

## 2 Methods

### 2.1 Participants

For this study 142 adolescent participants were recruited between ages 8 and 19 years (88 females and 54 males; *M* age = 14.48 years, *SD* = 2.76 years). The number of participants in each analysis is presented in the text and in the Supplementary Flow Chart (Fig. S1. The data for this study was collected as part of a larger longitudinal fMRI study on prosocial behavior. The participants were recruited through schools in the Netherlands. Only participants in households with a father and a mother were included. Even though this selection is not representative of all households, this decision was based on the practical reason that this was the most commonly occurring family structure. As such, the current study is a starting point for testing family relations and should be extended in future research to include other family structures such as two mothers or two fathers.

Participants were screened for MRI contra-indications and for a history of neurological and/or psychiatric disorders. All anatomical MRI scans were reviewed by a radiologist; no anomalous findings were reported. All participants above the legal age of 16 gave informed consent, with children below that age having both parents consenting before the start of the study. The study and all procedures were approved by the medical ethical committee of the University Medical Center.

### 2.2 Procedure

Before the scanning session, participants received general instructions and completed a practice run of the false-choice vicarious reward task to be played in the MRI scanner. In addition, all participants were first accustomed to the scanning experience in a mock-MRI scanner that simulates the scanning environment. After the scan-session of about one hour, the participants filled out questionnaires and played the prisoner’s dilemma game on a computer. The duration of the data collection session was around three hours in total. Participants received 20–40 euros as financial recompense depending on their age (<12 years 20 euros, 13–17 years 30 euros, >18 years 40 euros), plus an additional amount between 3.30–6.50 euros depending on their choices in other tasks. Out of this additional reward, 1 euro was specific to the vicarious reward task.

### 2.3 Materials

#### 2.3.1 COSY fMRI task

In the current study, an adapted version of the COSY (Charity or Self Yield) task was used to investigate the neural underpinnings of vicarious reward processes (Spaans et al., 2018b; Brandner et al., 2020). Before the scanning session, participants were instructed that they would play a game with their mother, their father, and a stranger, who was explained to be another anonymous participant of the study. In other words, this stranger manipulation was in terms of age closer to an unknown peer than an unknown adult. No additional information regarding the stranger’s gender, race or socioeconomic background was given. Parents were not present during the experiment. Participants were aware that they would receive a real monetary reward for themselves and their parents. This monetary reward was always 1 euro and identical for everyone based on the false-choice nature of the task. Similarly, they were told that the anonymous study participant would also receive rewards based on task outcomes for ecological validity. Accordingly, they also received some reward based on another participant’s trial outcome.

At the beginning of each trial, a jitter (black screen) was presented for 0–6.6 seconds (totaling to 15 % of the entire task time), followed by a fixation cross with a fixed duration of 500 ms. The stimulus presentation started with a screen presenting two curtains (one red, one blue) where participants were asked to choose one with a button press within 2 s). The outcome of this choice was random, i.e., participants could not influence the reward outcomes (see also Braams and Crone, 2017, for a similar heads-or-tails gambling format). A false-choice paradigm was selected to allow for a dissociation between reward processing from other processes that could confound the neural signal, such as reinforcement learning (Tamir and Hughes, 2018). Earlier studies showed that false-choice paradigms are better suited than passive paradigms due to perceived volition modulating reward-related activity in the striatum (Rao et al., 2008; Zink et al., 2004).

Once participants pressed a button, a hand icon holding onto a rope to open the curtains appeared next to the curtain the participant chose. After, on average (based on the reaction time of the participant), 2 s the hand icon pulled down the rope and revealed the monetary rewards behind the curtain of choice (the opening animation lasted for 700 ms, and was visualized using 15 distinct images of the curtains). The reward outcome was unknown to the participant during the response selection. Rewards were always displayed for the participant at the top of the screen with the outcome of one of the three targets visualized underneath. This feedback was presented for 2.3 s, which marked the end of each trial. The probabilities for all monetary outcomes were identical for all participants due to the false-choice nature of the paradigm. All participants received an equal number of all possible outcomes of the task. In case the participant did not press a button within the 2 s where he/she could choose one of the two curtains, an on-screen text informed that he/she was ‘too late’ for the duration equal to the regular length of a no-miss trial, and was followed by the next trial.

The task consisted of four conditions based on the outcomes presented: i) “*no gain*” condition entailing an outcome of 0 euros for both players, ii) “*both gain*” condition entailing 1 euro for both players, iii) “*other gain*” condition entailing 2 euros for the target and nothing for the participant, and iv) “*self gain*” condition entailing 2 euros for the participant and nothing for the other target. There were 15 trials of each of the four conditions and for each of the three targets, resulting in a total of 180 trials. The trials were randomized by both the identity of the three target partners as well as the monetary outcome conditions. The paradigm design was optimized for efficiency using opt-seq2 (Dale, 1999).

### 2.4 Survey and behavioral measures

#### 2.4.1 Pleasure from winning: survey

After the MRI session, participants were asked to indicate how much they liked winning money for themselves and for each of the three targets. All answers were given on a Likert scale ranging from 1 (‘did not like it at all’) to 7 (‘liked it a lot’). These data were available for n = 139 participants who completed this session (n = 3 exclusions, due to incomplete exit-interview).

#### 2.4.2 Cooperative behavior: behavior

**Fig. 1:**
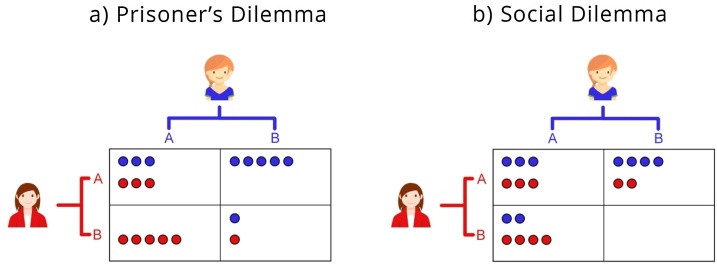
Design and differences in outcome between the a) Prisoner’s Dilemma Game (PDG) and b) The Social Dilemma Game (SDG). Avatars were chosen by the participants before the start of the session. Here, one trial for target ‘mother’ is shown for both the PDG & SDG. In both games, choice A depicts cooperation; choice B depicts defection. Differences in outcomes are due to the fact that the PDG is a zero-sum paradigm and the SDG a non-zero-sum paradigm.

To measure cooperative behavior towards different targets we employed the Prisoner’s Dilemma Game (PDG) and the Social Dilemma Game (SDG). The PDG is an operationalization of both social interaction and strategic behavior in classic game theory (Rand and Nowak, 2013). Within the PDG, the players have to make choices that benefit themselves or that benefit others, always dependent on the other players choices as well (Andreoni and Miller, 1993; Axelrod and Hamilton, 1981). As can be seen in Fig. 1A, if both players cooperate, they each receive the same payoff. In contrast, mutual defection results in a lower or no payoff. If, however, one player chooses to cooperate, while the other defects, then the defector receives the largest payoff possible, whereas the cooperator receives the lowest payoff possible. Participants played multiple one-shot trials without feedback (Frank et al., 1993). Players were instructed to be aware of the fact that the other (or target) they were playing this multiple one-shot game with could not see their choices at any point.

The reason for using a multiple one-shot game approach was to obtain a more robust measure of behavior. Moreover, participants were not expected to change their behavior over trials because there was no feedback or information on the other player’s choice. Reputation and trust building between players are therefore not possible and one’s strategy cannot be modified by the other players choices over time.

In addition to the classic PDG, which was a zero-sum game, participants also played a SDG, which was a snowdrift or non-zero-sum game. In game theory, zero-sum games are defined as strictly competitive situations in which one player’s payoffs are dependent on the other player’s payoff. In other words, one person’s gain is another player’s loss. In a social dilemma, on the other hand, both players’ gains can increase with cooperation, leading to a non-zero-sum context (see Fig. 1B). For both conditions, the strategic choices of cooperation and defection were labeled as A and B, respectively, in order to not invoke social desirability motivations and keep moral labels out of the decision process.

The behavioral tasks PDG and SDG were presented on a laptop computer, with two different dimensions (target/dilemma condition), for a total of six different conditions (target (3) x dilemma condition (2)). Participants played a total of 36 trials. The trials were randomized across targets (3) and context conditions (2). The participant, mother, father, and stranger were represented by flat-icon avatars chosen for each target by the player at the beginning of the experiment-session.

The instructions included a description of the structure of the game and the outcomes for both dilemma conditions (PDG/SDG). It was pointed out that they would play each trial separately with one of the three different targets. After the instructions, participants engaged in two practice trials, including 2 questions, testing whether the player had a good comprehension of the games. No participants had to be excluded from the analysis because of incorrectly answered practice questions. All decisions were coded as either ‘cooperate’ or ‘defect’. The total number of cooperations was averaged across trials for each condition. The task was administered to 92 participants, because programming of the task was completed a few weeks after the data collection started.

### 2.5 MRI acquisition

Participants were scanned using a Philips Achieva 3.0 T scanner using a 32-channel head coil. Following the localizer scan, first a T1-weighted structural scan was recorded (isotropic voxel size 1.1 mm^3^, RT =7.9 ms, TE = 3.5 ms, flip angle = 8 degrees, FOV = 250 mm, duration = 04:12 s) using a 3DT1 image sequence. Next, a T2* functional scan was performed (voxel size = 2.75 mm x 2.84 mm x 2.75 mm, RT = 2.2 s, TE = 30 ms, flip angle = 80 degrees, slice thickness, echo planar imaging [EPI], volumes = 3 × 188, number of slices = 38, FOV = 220 mm, duration = 3 × 07:09 s). Functional scans consisted of 3 runs with 188 volumes each and each run lasting 6 min; we discarded the first two scans to allow for stabilization of the signal. Participants were instructed to lie still in the scanner and were constantly monitored through a camera system. Furthermore, head movements were restricted by using foam triangles to fill available empty space between participants’ heads and the head coil.

### 2.6 Pre-processing

Neuroimaging data were pre-processed and analyzed using SPM8 (Wellcome Trust Centre for Neuroimaging, London). For pre-processing, we corrected all functional scans for slice timing and excessive head motion (6 parameters). Following the Coregistration of the T2* with the structural scan we resampled all volumes to the resolution of 3 mm^3^. Normalization to an anatomical atlas was based on MNI305 (Cocosco et al., 1997). Finally, we used an isotropic Gaussian kernel (6 mm FWHM) to spatially smooth the data.

### 2.7 fMRI analysis

We modeled the fMRI time-series with the hemodynamic response function (HRF) convolution and with the outcome timings of each condition. This allowed us to create contrasts to be used during the first-level-analysis. We modeled the first moment of reward outcome presentation (image 7 within the opening animation) as a null duration event for each of the four outcome conditions: ‘no gain’, ‘both gain’, ‘self gain’, and ‘other gain’, for each of the three targets resulting in 12 conditions in total. All of these events were time-locked with a zero-duration to the exact moment that participants were able to see the first image of the monetary reward (i.e., the seventh frame of the curtain-opening animation). Trials without a response were coded as invalid and excluded from further analysis. A general linear model (GLM) was created using all twelve conditions, along with motion regressors and a high pass filter of 120 Hz. The (least square) parameter estimates (beta-weights) of the best-fitting canonical HRF for each condition were used in pairwise contrasts. These contrasts were then used in the random-effects group-analysis. Three participants had to be excluded because of mask distortions and an additional 14 participants had to be excluded because of movement larger dan 3 mm in any direction, resulting in n = 17 exclusions in total (for a detailed diagram showing all exclusions, see Supplementary Fig. S3). Whole brain comparisons, based on in total 125 participants, are presented on Neurovault: https://neurovault.org/collections/JNKBCYPJ/.

### 2.8 Region of interest (ROI) selection

To investigate the neural activation patterns of vicarious rewards for parents and strangers, we performed NAcc ROI analysis using the *Marsbar* toolbox in SPM8 (Brett et al., 2002). This bilateral region in the ventral striatum was chosen for its robust role in reward processing and was based on a predefined anatomical ROI of the left and right NAcc as extracted from the Harvard-Oxford subcortical atlas and thresholded at 40 %; for details see (Braams et al., 2015). The ROI mask consists of 28 voxels for the left NAcc (coordinates left: x = −9.57, y = 11.70, z = −7.10) and 26 voxels for the right NAcc (coordinates right: x = 9.45, y = 12.60, z = −6.69). Following the ROI selection, we extracted parameter estimates for the analysis. None of the results showed differences between the left and right NAcc, therefore all the analyses were performed by collapsing across both hemispheres.

To investigate the neural activation for winning for self and others, we used the *no gain* condition as the baseline in the task. To test whether there were differences in NAcc activation between the three targets in the no-gain condition, we performed a target (3) ANOVA for no-gain outcomes. The difference between the three no-gain conditions was not significant *F*(2,250) = .36, *p* = .70. Therefore, we collapsed across the no-gain conditions and used average NAcc activation in the no-gain conditions as a baseline in all analyses. In examining the neural activation for winning for the self, we examined the *self gain* – *no gain* contrast; for neural activation when winning for the target, we examined the *other gain* – *no gain* contrast and finally, for the mutual condition we examined the *both gain* – *no gain* contrast.

As part of an exploratory analysis one additional ROI was investigated. Based on aggregated fMRI data from Neurosynth (all studies that matched the keyword “reward”) we created a sphere with 10 mm radius with the center at (x = 2, y = 44, z = -4) in the vmPFC (see Supplementary S4). All additional steps are identical to the ones describe above for the NAcc ROI analysis.

## 3 Results

### 3.1 Pleasure from winning: survey results

**Fig. 2:**
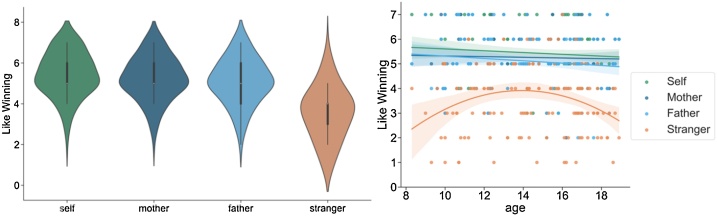
(A) Pleasure ratings when winning rewards for targets self, mother, father and stranger. Self-ratings were higher than ratings for all other targets. Stranger-ratings were lower than ratings for all other targets. Mother-ratings were higher than father-ratings. (B) Pleasure ratings when gaining for targets self, mother, father and stranger across age. Stranger-ratings showed a quadratic peak with highest ratings in mid-adolescence.

Our confirmatory hypothesis (*hypothesis #13*) predicted that self-reported pleasure of winning monetary rewards would be higher for family members (mother, father) than for the stranger target. To test this hypothesis, we conducted a one-way ANOVA for the outcome variable ‘pleasure from winning’ for the four targets: self, mother, father, stranger (n = 139). The result shows a significant difference between groups, *F*(3,414) = 109.97 *p* < .001 (see Fig. 2). Pair-wise LSD comparison tests showed that pleasure from winning for self was significantly higher than pleasure from winning for father (Self-Father; MD = 0.28, *p* = .008, Self-Stranger; MD = 1.89, *p* < .001), but not for mother (Self-Mother; Mean difference (MD) = 0.12, *p* = .202). Pair-wise LSD comparison tests revealed a small but significant higher pleasure from winning for mother than father (MD= 0.158, *p* = .023), and a significantly higher pleasure from winning for mother than stranger (MD = 1.77, *p* < .001) and for father than stranger (MD = 1.61, *p* < .001).

Next, we conducted an exploratory analysis to investigate the developmental differences in pleasure from winning for each of the different players. Age-linear and age-quadratic were added as a covariate to the one-way ANOVA. The interaction between target and age-quadratic was significant, *F*(3, 411) = 5.36, *p* = .001. Separate linear regression analyses were conducted for each of the 4 possible targets (self, mother, father, stranger) separately. Age^2^ was added to examine quadratic developmental associations with pleasure from winning as the outcome variable. This analysis showed a significant fit only for the pleasure winning for stranger condition, *F*(1,137) = 7.60, *p* = .007, whereas pleasure winning for self, mother and father did not reveal developmental differences. As can be seen in Fig. 2, the inverted curve for stranger peaked at around 14-years-old with lower scores observed between 8–13 and 15–19.

### 3.2 Neural responses to reward: NAcc results

#### 3.2.1 Self-gain

The pre-registered hypothesis (*hypothesis #15*) predicted higher activation in the NAcc ROI for the self-gain conditions contrasted with the no-gain condition (n = 125). For this analysis, an average self-gain condition was computed across the three targets. The self-gain versus no-gain comparison showed significantly higher NAcc activity for rewards for the self, compared to not gaining rewards (no-gain), *F*(1,124) = 55.96, *p* < .001. We expected this activity to be stronger in mid-adolescence (*hypothesis #15*). However, adding age-linear (*F*(1,123) = .11, *p* = .74) and age-quadratic (*F*(1,123) = .94, *p* = .33) as a covariates to the ANOVA did not result in significant main or interaction effects of age.

#### 3.2.2 Both-gain and other-gain

We further tested for differences in the NAcc activation between the self-gain, both-gain and other-gain conditions (compared to the baseline condition of average no-gain) for the three targets. We expected that neural responses associated with vicarious rewards would be larger for the mother and father conditions than for the stranger condition (*hypothesis #16*).

**Fig. 3:**
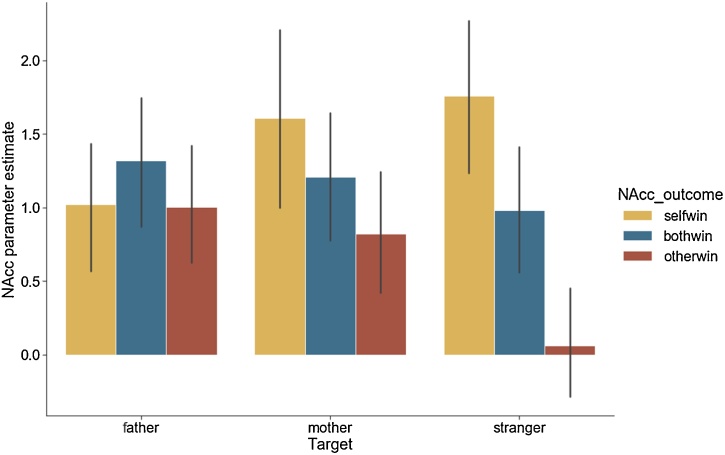
NAcc responses for self and vicarious rewards for the three targets. A target x condition interaction revealed that vicarious rewards were significantly lower for strangers. All three conditions are contrasted against the baseline of the no-gain condition.

We conducted a 3 (target) x 3 (self-gain, both-gain, other-gain) repeated measures ANOVA for NAcc ROI activation. There was a main effect for condition (*F*(2,248) = 9.42, *p* < .001), no main effect of target (*F*(2,248) = 1.29, *p* = .27), and a significant target x condition interaction (*F*(4,496) = 4.86, *p* = .001; see Fig. 3).

Post hoc ANOVAs were conducted for each condition separately. The self-gain and both-gain conditions did not result in a significant effect of target (*F*(2,248) = 2.77, *p* = .065 and *F*(2,248) = .57, *p* = .56, respectively), whereas the other-gain condition resulted in a significant effect of target (*F*(2,248) = 9.53, *p* < .001). Pairwise LSD comparisons confirmed that neural response in NAcc was significantly higher for mother and father than for stranger (both *p*’s <.002). Mother and father did not differ significantly from each other (p = .56). A one-sample *t*-test further revealed that other-gain in the stranger condition was not significantly different from zero (*t*(124) = .49, *p* = .69), suggesting that this condition resulted in no vicarious reward responses in the NAcc (detailed figures differentiating target and condition across the developmental age range can be found in the Supplementary Fig. S2).

We expected that vicarious NAcc responses would be higher in mid-adolescence (hypothesis #16). For this purpose, age-linear and age-quadratic were added as covariates to the condition ANOVAs for each target separately. No effects of age were observed for any target (see Supplementary Fig. 2).

#### 3.2.3 Survey pleasure ratings and NAcc responses

Finally, we explored whether NAcc responses to vicarious rewards were related to pleasure from winning by examining the correlations between pleasure ratings and neural activity following vicarious gains (both-gain and other-gain). As can be seen in Table 1, correlations were significant for other-gain for mothers and fathers, but not for strangers. These correlations remained significant when controlling for age and confirm a positive relation between neural responses to vicarious rewards and self-report of pleasure experienced from these vicarious rewards.

**Table 1:** Correlations between neural activity in NAcc in the vicarious gain conditions (relative to the both no-gain baseline) and self-report ratings of pleasure experienced from these respective vicarious rewards (n = 124).

NAcc activity	Pleasure Mother	Pleasure Father	Pleasure Stranger
Both-gain	*r* = .091,	*r* = .134,	*r* = .112,
	p = .32	p = .14	p = .21
Other-gain	*r* = .252,	*r* = .277,	*r*=−.063, p = .48
	p = .005	p = .001	

### 3.3 Prisoner and social dilemma behaviors

The prisoner’s dilemma game was played by the participants with three targets (mother, father, stranger) across two reward-contexts (classic prisoner’s dilemma and social dilemma). We first present exploratory task and age effects on cooperative behavior, followed by a confirmatory test of the relation between vicarious NAcc activity and cooperation (n = 92).

In the first analysis we compared how often participants cooperated in each of the six conditions. A target(3) x context(2) repeated measures ANOVA was performed, which resulted in a main effect of target (*F*(2,182) = 16.84, *p* < .001) and a main effect of context (*F*(1,91) = 54.65, *p* < .001); the target x context interaction effect was not significant (*F*(2,182) = 2.30, *p* = .103). We explored the pair-wise comparisons for the main effect of target using a post-hoc LSD pairwise comparison tests. For the target conditions participants cooperated less with strangers than with mothers (MD = −0.88, *p* < .001), and also less with strangers compared to fathers (MD = −0.67, *p* < .001). The difference between mothers and fathers was not significant (MD=−.20, *p* = .08). The main effect of context revealed that participants cooperated more in the social dilemma than in the prisoner’s dilemma (MD = 1.46, *p* < .001).

#### 3.3.1 Age-comparisons Prisoners Dilemma task

**Fig. 4:**
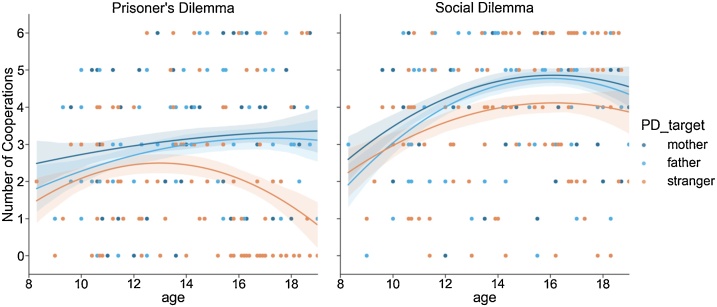
Age patterns in cooperation in the Prisoners Dilemma (PDG) and Social Dilemma (SDG) game for targets mother, father and stranger. A. Cooperation showed an age x target interaction in the Prisoners Dilemma game. B. Cooperation showed a main quadratic age effect in the Social Dilemma game.

We investigated the developmental patterns of age-related changes in cooperation in the prisoner’s dilemma game by including age as a covariate in the one-way ANOVA with target (3 levels) as a within subjects factor. This analysis revealed a significant target x age interaction, *F*(2, 180) = 4.68, *p* = .01. This interaction was followed up by separate post hoc ANOVAs to compare targets directly. Cooperative behavior was not significantly different between mothers and father (*F*(1, 90) = .27, *p* = .61). However, target x age interactions were observed when comparing mothers and strangers (*F*(1, 90) = 5.00, *p* = .028) and when comparing fathers and strangers (*F*(1, 90) = 6.62, *p* = .012). Visual inspection of Fig. 4A shows that with increasing age (around 14), participants differentiated more between in-group (i.e., family) and out-group members (i.e., strangers) in their cooperative behavior.

#### 3.3.2 Age-comparisons social dilemma task

The same target (3) ANOVA analysis with Age as covariate for the social dilemma revealed both a main effect of age-linear (*F*(1,90) = 9.90, *p* = .002), and of age-quadratic (*F*(1,90) = 7.53, *p* = .007), but no significant target x age interactions. Thus, social dilemma collaboration showed a significant linear/quadratic fit suggesting a rapid increase in collaboration between early and mid-adolescence leveling off in late-adolescence/early adulthood (see Fig. 4B).

#### 3.3.3 Relation winning pleasure, NAcc and prisoner’s dilemma choices

We expected to find positive relationships between NAcc activation for other-gain conditions and cooperative behavior towards the respective targets (hypothesis #17) (n = 91). Correlation analyses showed that this hypothesis was not confirmed. We found no correlation between cooperation in either the Prisoner’s or the Social Dilemma games and vicarious (other-gain) NAcc activity for any of the three targets.

We observed that cooperation in the PDG & SDG was negatively correlated with NAcc activity in the self-gain condition when playing with the mother (see Table 2). In other words, higher NAcc activity for self-gain (at the expense of mothers) was associated with less cooperation with mother in both the PDG and the SDG (see Table 3). Moreover, pleasure from winning for strangers was positively correlated with PDG cooperation for strangers. Finally, pleasure from winning for self was negatively correlated with cooperation in the SDG for stranger (detailed figures differentiating between PDG and SDG across targets can be found in the Supplementary Fig. S2).

**Table 2:** Correlations between pleasure from self and vicarious (other) gaining, neural activity in NAcc in the vicarious gain conditions (relative to the both no-gain baseline) and Prisoners Dilemma cooperation (separated by target). All correlations remain significant when controlling for age. Significant correlations in bold.

	PD Mother	PD Father	PD Stranger
Self-Pleasure	*r*= −.149, p = .16	*r*= −.122, p = .25	*r*= −.092, p = .39
Vicarious Pleasure	*r*= −.001, p = .99.	*r* = .141,	***r* = .312,**
		p = .18	***p* = .003**
NAcc Self-gain	***r*= −.296,**	*r*= −.104, p = .33	*r* = .003,
	**p = .004**		p = .98
NAcc Both-gain	*r*= −.025, p = .82	*r*= −.174, p = .10	*r* = .045,
			p = .67
NAcc Other-gain	*r*= −.035, p = .74	*r*= −.130, p = .22	*r*= −.136, p = .20

**Table 3:** Correlations between pleasure from self and vicarious (other) gaining, neural activity in NAcc in the vicarious gain conditions (relative to the both no-gain baseline) and Social Dilemma cooperation (separated by target). All correlations remain significant when controlling for age. *p < .05, not Bonferroni corrected. Significant correlations in bold.

	SD Mother	SD Father	SD Stranger
Self-Pleasure	*r*= −.205,	*r*= −.232,	***r*= −.283,**
	p = .052	p = .03*	***p* = .007**
Vicarious Pleasure	*r*= −.208,	*r*= −.255,	*r* = .240,
	p = .049*	p = .03*	*p* = .02*
NAcc Self-gain	***r*= −.331,**	*r*= −.088, p = .41	*r*= −.111, p = .29
	***p* = .001**		
NAcc Both-gain	*r*= −.129, p = .22	*r*= −.011, p = .92	*r*= −.106, p = .32
NAcc Other-gain	*r*= −.044, p = .68	*r*= −.098, p = .36	*r*= −.196, p = .06

### 3.4 Exploratory analysis: vmPFC response to reward

**Fig. 5:**
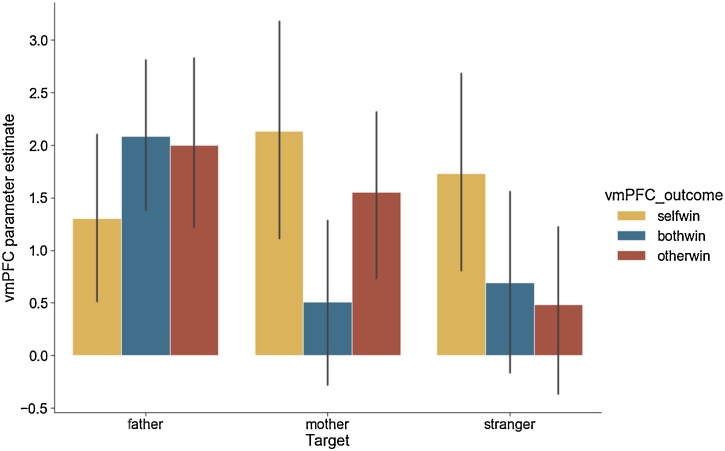
vmPFC responses for self and vicarious rewards for the three targets. All three conditions are contrasted against the baseline of the no-gain condition.

To provide a more complete picture of the vicarious reward processes one additional exploratory vmPFC ROI analysis was conducted. A repeated-measures 3 × 3 (target x condition) ANOVA was performed (see also Fig. 5). No target (*F*(2,258) = 2.73, *p* = .06) or condition effect was found (*F*(2,258) = 1.22, *p* = .29). However, we did find an interaction effect between target and condition (*F*(4,516) = 4.37, *p* < .01). Post hoc ANOVAs were also conducted for each of the 3 conditions separately (mirroring the NAcc analysis above). The self-gain condition did not result in a significant effect of target (*F*(2,258) = 1.21, *p* = .29). The both-gain condition and the other-gain condition resulted in significant effect of target results (*F*(2,258) = 4.91, *p* < .001 and *F*(2,258) = 4.91, *p* = .001, respectively). Follow up paired *t*-test analyses comparing the targets showed that for both-gain, vmPFC activity was significantly higher for father compared with mother (*t*(274) = 2.87, *p* = .004) and stranger (*t*(274) = 2.44, *p* = .01). In contrast, for other-gain, vmPFC activity was significantly higher for father compared with stranger (*t*(274) = 2.62, *p* < .001), whereas the mother condition did not differ significantly from father (*t*(274) = 0.76, *p* = .44), or stranger (*t*(274) = 1.85, *p* = .06).

Furthermore, we analyzed whether vmPFC vicarious reward response was associated with identical responses in the NAcc for all three targets. Significant correlations between vmPFC and NAcc ROI responses were found for the OtherWin condition for target mother (*r* = .61, *p* < .001), father (*r* = .66, *p* < .001), and stranger (*r* = .47, *p* < .001). See Fig. S5 in the Supplementary materials for details.

## 4 Discussion

In this study, we investigated the relationship between neural vicarious reward processing and cooperative behavior for family members and strangers across adolescence. Vicarious reward responses were measured through self-reports of pleasure from winning for others and NAcc responses during a false-choice fMRI task (Brandner et al., 2020) that was played with three targets: mothers, fathers, and strangers. These vicarious reward measures were related to cooperative behavior assessed by the prisoner’s dilemma (PDG) and social dilemma (SDG) games. We confirmed higher self-reported enjoyment for winning for self and parents compared with strangers (*hypothesis #13*). As expected, neural activity in NAcc was higher for self-gains versus no-gains (*hypothesis #15*). We also confirmed higher NAcc activation when winning for close others (parents) compared with strangers (*hypothesis #16*). Contrary to our hypotheses (*#15 and #16*), no age-related peaks were observed in any of the self or vicarious reward data. Contrary to our expectations, cooperative behavior was not correlated with vicarious reward processing at the neural or behavioral level (*hypothesis #17*). However, exploratory analyses revealed several novel findings, showing that with increasing age, participants showed stronger differentiation between winning pleasure and cooperation for in-group versus out-group members.

### 4.1 Neural rewards for self and others

Consistent with prior research, we observed that the NAcc shows stronger activity to self-gain rewards relative to no-gain for either target (Spaans et al., 2018a). These findings extend prior findings showing that when self-gain is limited to self and does not extend to the other target, there is a robust NAcc reward activity (Christopoulos and King-Casas, 2015). Prior studies provide preliminary evidence of NAcc vicarious reward activity (Brandner et al., 2020; Morelli et al., 2015). Here, we showed that in adolescence, vicarious reward activity was observed for mothers and fathers, but not for strangers (see also Braams and Crone, 2017). Interestingly, vicarious reward activity for mothers and fathers did not differ from reward activity for self-gain, suggesting that family members are an important source of vicarious joy (Telzer, 2016).

Self-reports of winning pleasure confirmed these neural patterns, showing that winning pleasure was lower for strangers relative to the other targets. This finding is consistent with prior studies showing that gaining for unknown others results in lower winning pleasure (Braams et al., 2015; Brandner et al., 2020). Furthermore, a positive correlation was found between self-reported vicarious joy for both parents and NAcc vicarious reward activation for mothers and fathers. This provides further support for the validity of this paradigm in measuring vicarious reward processing in the ventral striatum and its relationship with subjective feelings of vicarious joy for a close other. Also, within the in-group targets, there were subtle differences in self-report winning pleasures. Winning pleasures were highest when gaining for self and mothers and slightly but significantly lower for fathers. In a prior study, we observed the same behavioral effect of small but significantly lower winning pleasure for fathers (Brandner et al., 2020). This difference was associated with neural differences in reward activity between mothers and fathers, such that neural activity to self-gain was lower when this implicated that fathers did not gain anything (Brandner et al., 2020). In the current developmental study, the trend was in the same direction but this difference was not significant (*p* = .06), possibly suggesting that lower self-gain activity when the other target is a father compared to a mother increases in adulthood.

Finally, we demonstrated developmental differences in winning pleasure such that winning pleasure for strangers was higher in mid-adolescence relative to childhood and adulthood, combined with larger family-stranger differentiation in late adolescence. These findings fit with prior behavioral studies showing that friend-stranger differentiation in prosocial behavior also increases in late adolescence (Güroğlu et al., 2014). Contrary to expectations, these differences were not associated with developmental changes in neural activity to self-gain and vicarious gain. Possibly, the neural peak in mid-adolescence for self-gains is only observed when these rewards are not presented in a social comparison context (see also Spaans et al., 2018a). Conversely, the stability and changing nature of the relationship with parents but also strangers might obscure an age effect. A recent study looking at ventral striatum activity for vicarious reward with best friends found quadratic age effects only in stable friendships and not in unstable ones (Schreuders et al., 2021).

Alternatively, developmental differences may be observed in neural signals in other brain regions, including the vmPFC (Blakemore and Mills, 2014). We explored activity in vMPFC as a core region associated with reward processing, as part of the mesolimbic ‘reward circuit’ (Koban et al., 2021). The pattern of activity was similar to the NAcc, showing that the vmPFC is more active when processing vicarious rewards for parents than for strangers. There were also subtle differences in activation patterns, such that mutual gain led to stronger activity in the vmPFC for fathers than for mothers, consistent with our prior findings in adults (Brandner et al., 2020). The vmPFC is also interpreted as a convergence zone linking reward, self and mentalizing processes (Crone and Fuligni, 2020). Therefore, future studies should examine vicarious rewards for multiple targets in these associated brain networks as well.

### 4.2 Cooperation with family and strangers

Consistent with prior developmental studies, cooperative behavior increased with age. This finding was observed for cooperative behavior as assessed by both the PDG and the SDG. Cooperation was also generally higher when playing a non-zero-sum SDG independent of the target. Most interestingly, cooperative behavior towards parents and strangers did not differ in the SDG (both rose with age) but showed a robust divergence with increasing age in the PDG. In the zero-sum PDG, cooperation with strangers aligned with those for parents up approximately to the age of 13−14-years after which cooperation for strangers decreased. This provides evidence that consistent with the results for winning pleasure, with increasing age adolescents differentiate more between in-group and out-group members (Güroğlu et al., 2014; Telzer, 2016; van de Groep et al., 2020). Our study is the first to investigate this in the parent-child relationship context (with both mother and father).

Contrary to expectations, vicarious reward activity was not related to cooperative behavior in the Prisoner’s Dilemma and Social Dilemma games. This finding may suggest that mechanisms other than vicarious reward processing are involved in deciding whether to cooperate with others, such as perspective-taking (Dumontheil et al., 2010) or deviating from strict equity norms (Güroglu et al., 2014; Meuwese et al., 2015). Interestingly, we observed a negative correlation between cooperative behavior towards the mother and NAcc activity for rewards for the self when playing with the mother. One explanation could be that higher reward activation for oneself is related to less cooperative behavior with that same target (Meuwese et al., 2018). Another possible explanation could lay in the developmental reorientation during adolescence. In this critical phase of development, adolescents shift their interpersonal focus away from the home and family environment and more towards the external world. This shift is accompanied by tensions in the adolescent process of gaining autonomy and a self-identity independent of the primary care-takers, often mothers, at home. Future research is definitely necessary because in the current sample the observed effect did not extend to fathers or strangers.

### 4.3 Limitations and future directions

Some limitations of the current study should be noted. The false-choice fMRI COSY task was chosen with the intention to exclude confounds of behavioral choices (Spaans et al., 2018b; Brandner et al., 2020). Even though choices were made on each trial, the participants were not able to affect the outcome during this task. This design provides a trade-off between design simplicity and allowing for player engagement. The advantages of this experimental design are the exclusion of confounding processes as observed in previous donation tasks. Classic donation tasks are known for their skewed results with most participants answering on one end of the scale, leading to low variance and underpowered analyses. With the current COSY task we were able to control for this and as a result, assured high statistical power across all conditions. A limitation is that the focus on vicarious joy (as implemented with vicarious reward processing) limits the interpretation of results in combination with behavioral data. Vicarious joy by itself is not an action-oriented social emotion, it does not lead to motivated behavior. In other words, it is a passive appreciation of another person’s positive experience. Therefore, vicarious joy may be limited as a motivational force driving prosocial cooperative behavior. A further limitation is the homogeneity of the adolescent sample. Generalizability of our results for countries and cultures with different social norms as well as the perceived importance of individuality versus group-identity might be limited.

Another point of limitation involves the implementation of the stranger condition. The participant was instructed that the stranger is another participant in the study, all of whom were between 8–19 years old. This allowed for the uncontrolled variable of the participants age and how old they imagined the stranger to be, resulting in several imagined age and power-differentials combinations. This combinatorial variable might be a reason for the quadratic age effect in the experienced joy survey results, which was only observed in the stranger condition. Future studies could improve on this by being more precise in their definition of the stranger target. For instance by introducing them as another study participant of the same age.

Additionally, and in a similar fashion, one final limitation revolves around the adolescents’ expectation when it came to the monetary reward won for their parents. Some participants might implicitly or even explicitly have assumed that money won for their parents will somehow trickle down to themselves. Future studies, should take this into account by collected an exit-interview question assessing the participants assumptions of monetary distribution. Furthermore, adding another in-group target that is not the parents (i.e. best friend) could allow for a more nuanced picture and differentiation based on monetary expectations within families that don’t apply to other in-groups.

While our results show the utility of our experimental design to investigate vicarious joy as well as cooperative behavior in a developmental sample, some suggestions for further improvements are discussed. Primarily, future research should include a broader spectrum of social emotions. To elucidate the relationship between participants and targets as well as their emotional valence we suggest including conditions of compassion, envy, and schadenfreude (Steinbeis and Singer, 2014). To realize a more detailed distinction between social targets, the inclusion of peers or friends, especially during adolescent development, might prove to be of value in future studies.

## 5 Conclusions

The unique contribution and aim of this study were to highlight developmental differences in adolescence for vicarious reward and cooperative behavior based on the relationship to the target. We were able to provide evidence for selective processing of vicarious reward in the NAcc based on the relationship to a target. In addition, we showed that cooperation increased with age in non-zero-sum context. A distinction between close and distant others in cooperative behavior became apparent in a zero-sum prisoner’s dilemma condition, showing that with increasing age adolescents differentiate more between in-group and out-group members in winning pleasure and cooperation.

Our study shows the value of using a combination of neural, behavioral, and questionnaire data to investigate complex social constructs in a developmental population. By combining a task-fMRI paradigm with behavioral experiments and questionnaire data we were able to reveal associations between fundamental vicarious reward processing in the striatum with self-reported enjoyment. Together, the results provide important next steps towards understanding ingroup and outgroup considerations in vicarious joy and cooperative behavior.

## Data & code availability statement

All data used for analysis within the manuscript has been made openly available on appropriate repositories.

Statistical group maps (whole-brain fMRI results) have been shared and uploaded to Neurovault.org (private link: https://neurovault.org/collections/JNKBCYPJ/).

fMRI ROI results and behavioral measurements used for statistical analysis, included the python code (in form of a jupyter notebook) for the statistical analysis & figure creation has been made available on figshare: https://figshare.com/s/87a5611fb53cfd8f1f21.

## Declaration of Competing Interest

The authors declare that they have no known competing financial interests or personal relationships that could have appeared to influence the work reported in this paper.
