# Supplementary material for: Happy for Us not Them: Differences in neural activation in a vicarious reward task between family and strangers during adolescent development
Source: Dev Cogn Neurosci. 2021 Jun 30;51:100985. doi: 10.1016/j.dcn.2021.100985 (PMC8319462; doi:10.1016/j.dcn.2021.100985)
Supplement: Supplementary file 1 [file mmc1.docx]

**Supplementary material**


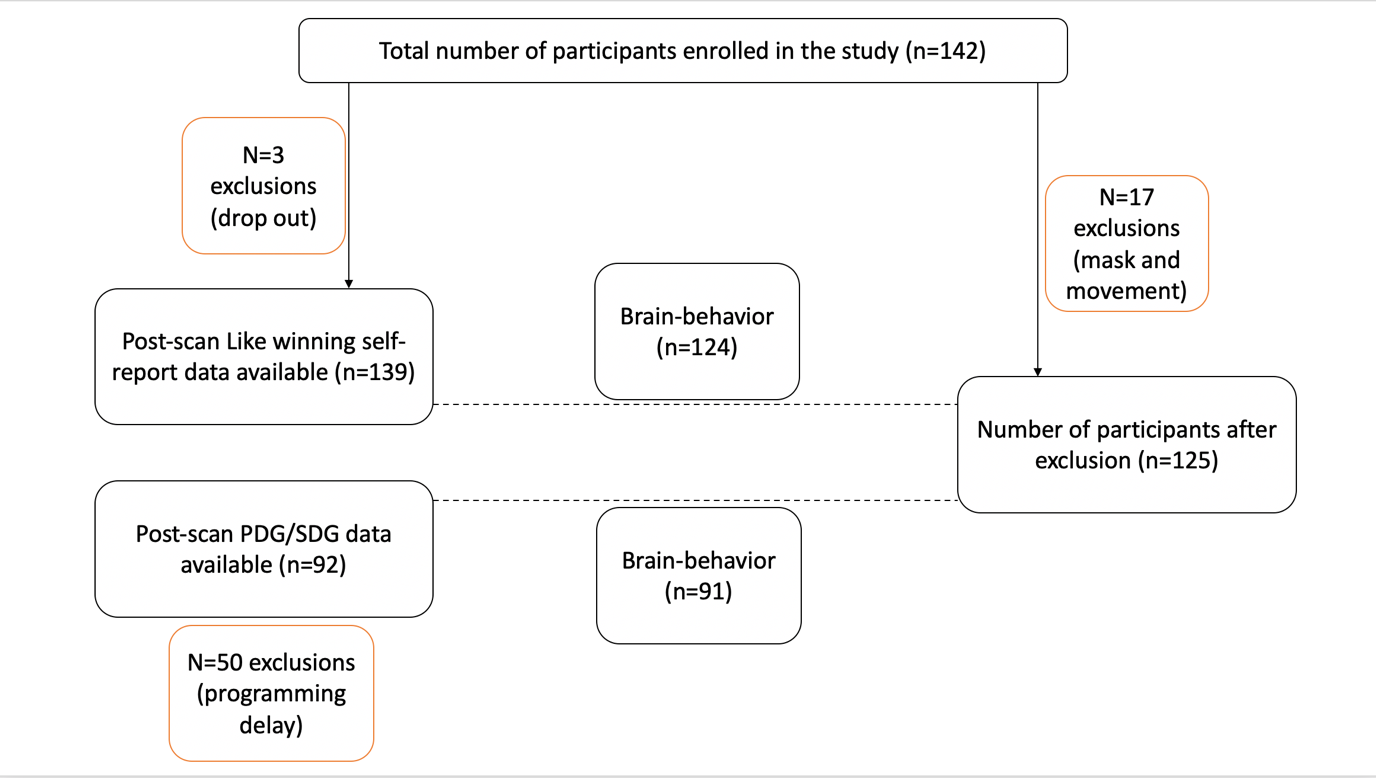


Figure S1: Participant flow-chart of the different analysis steps and sample sizes used within the result section.

We expected to find higher NAcc activation for each parent if questionnaire results point to a stronger emotional relationship within the family (assessed using the FACES IV questionnaire, (Olson, 2011)) (hypothesis #18) . The data did not support this hypothesis. Correlation between Family Cohesion and NAcc ROI activation for mother (*r* = 0.07, *p* = .43) and father (*r* = 0.03, *p* = .70) were non-significant.

We also expected to find a positive correlation between NAcc activity for the other-gain > self-no-gain contrast (for stranger) for participants with higher prosociality scores as measured by the Prosocial Tendencies Measurement Revised scale (Carlo, Hausmann, Christiansen, & Randall, 2003) (Hypothesis #19). This hypothesis was not confirmed (*r* = -0.03, *p* = .70). We, additionally expected to find a positive correlation between NAcc activation for the stranger in the OtherWin> self-no-gain contrast and self-reported perspective-taking skills and empathic concern (hypothesis #20). Results revealed no significant association between NAcc and perspective taking (*r* = 0.07, *p* = .40) or empathic concern (*r* = - 0.06, *p* = .48).

*
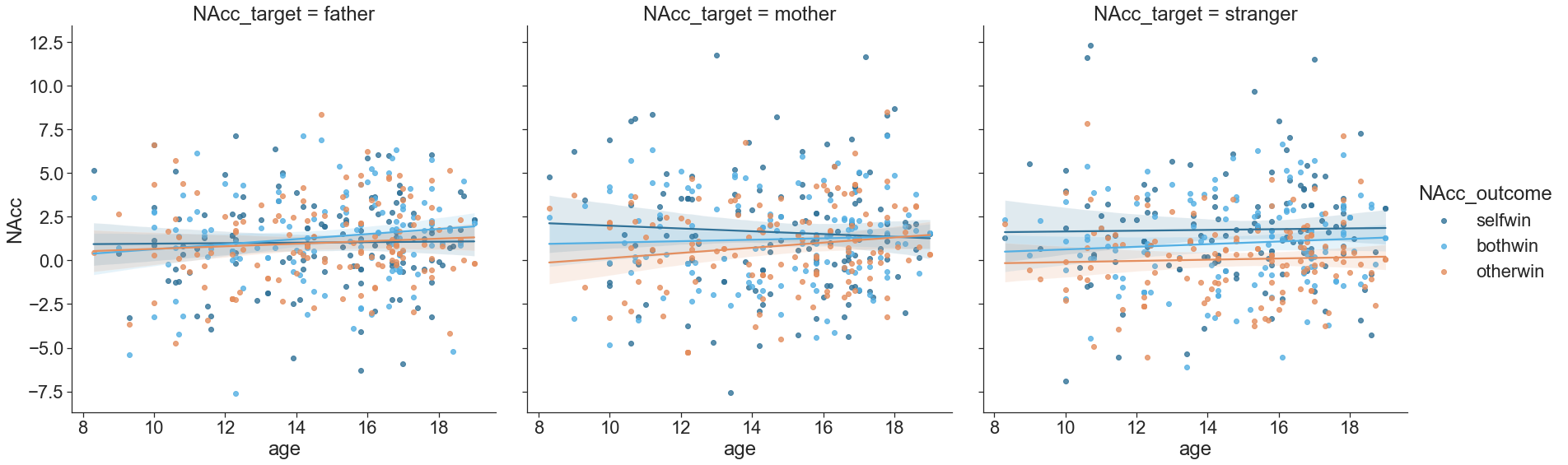
*

Figure S2: NAcc ROI activation as a function of age based on the target (column) and the condition (color).


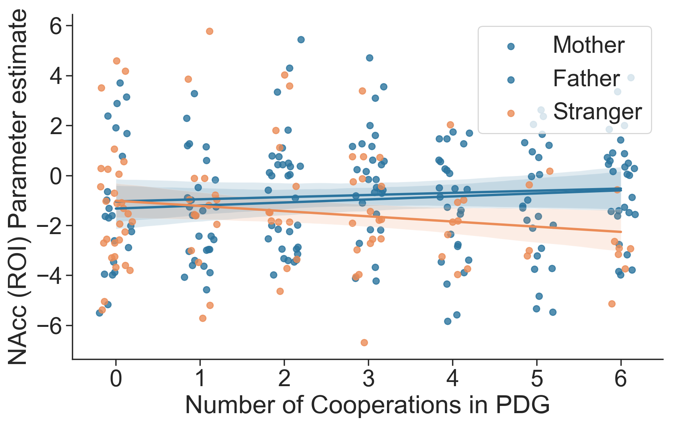

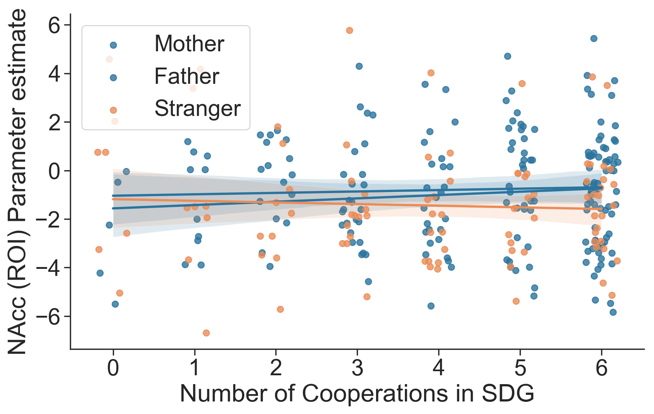


Figure S3: Scatterplot of NAcc ROI activation (for Vicarious reward condition OtherWin) and number of cooperations for the PDG and the SDG for all targets (colors).


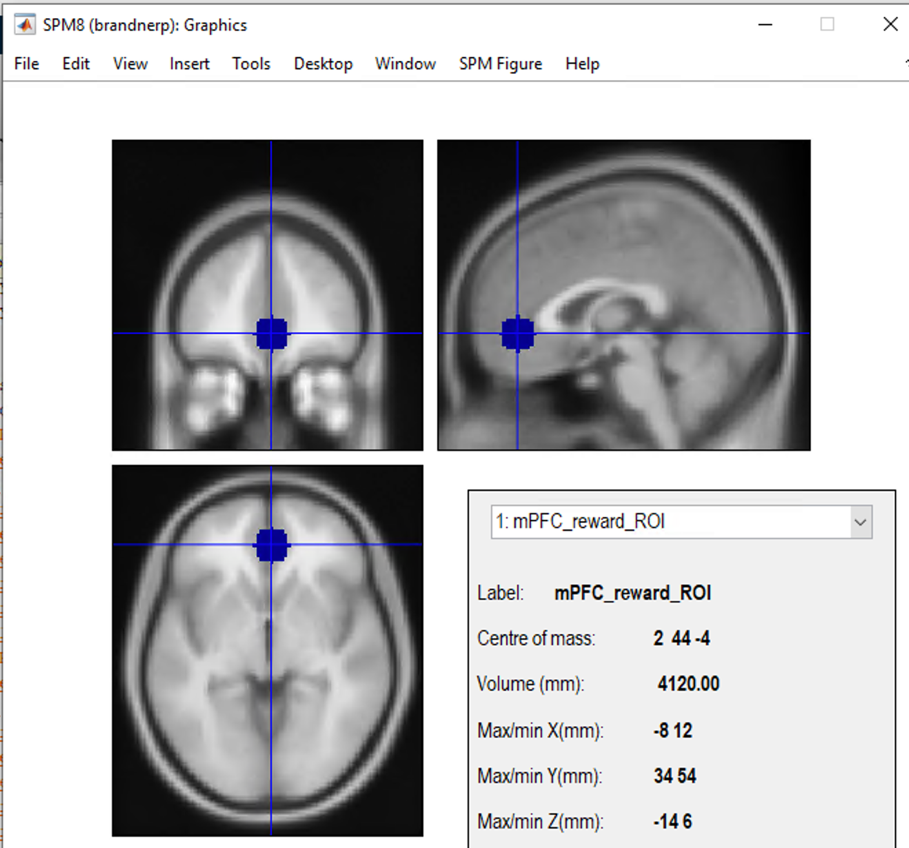


Figure S4: vmPFC ROI creation using SPM8 and the Marsbar toolbox. Center of the sphere was at voxel x = 2, y = 44, z = -4. A 10 mm radius was drawn around it.


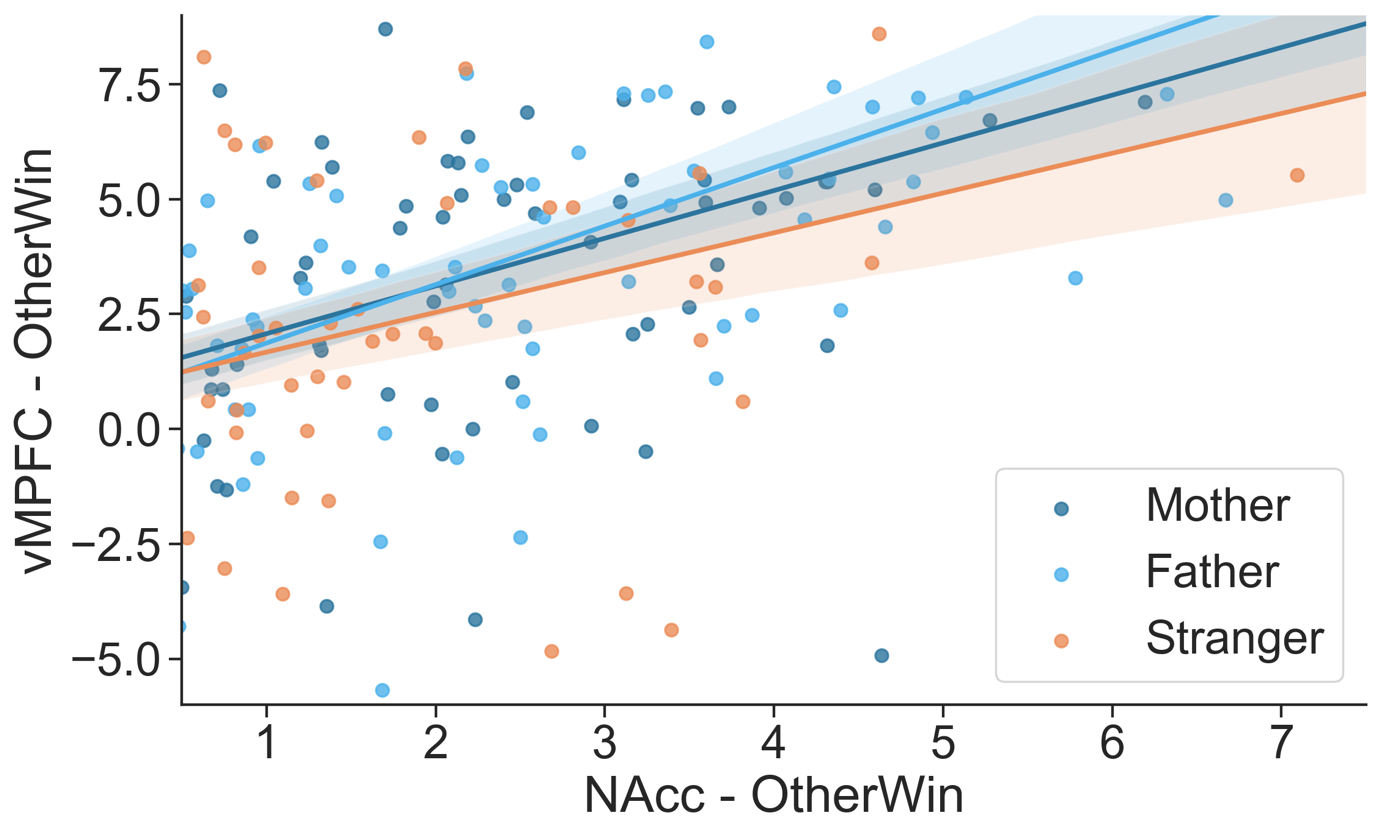


Figure S5: Correlation between vmPFC and NAcc for the vicarious reward condition (OtherWin) for all three targets . Significant and large correlation effects for all three targets (r range from 0.5 to 0.65).
